# Ethyl 2-(5-meth­oxy-2-methyl-1*H*-indol-3-yl)acetate

**DOI:** 10.1107/S1600536813018618

**Published:** 2013-07-13

**Authors:** Shaaban K. Mohamed, Joel T. Mague, Mehmet Akkurt, Alaa A. Hassan, Mustafa R. Albayati

**Affiliations:** a Chemistry and Environmental Division, Manchester Metropolitan University, Manchester M1 5GD, England; b Chemistry Department, Faculty of Science, Mini University, 61519 El-Minia, Egypt; c Department of Chemistry, Tulane University, New Orleans, LA 70118, USA; d Department of Physics, Faculty of Sciences, Erciyes University, 38039 Kayseri, Turkey; e Kirkuk University, College of Science, Department of Chemistry, Kirkuk, Iraq

## Abstract

In the title compound, C_14_H_17_NO_3_, the nine-membered 1*H*-indole ring system is essentially planar [maximum deviation = 0.019 (1) Å]. In the crystal, mol­ecules are linked *via* N—H⋯O hydrogen bonds, forming chains along [001]. These chains are linked *via* C—H⋯O hydrogen bonds and C—H⋯π inter­actions, forming a two-dimensional network lying parallel to the *ac* plane.

## Related literature

For medicinal applications of the drug indomethacin (systematic name: 2-{1-[(4-chloro­phen­yl)carbon­yl]-5-meth­oxy-2-methyl-1*H*-indol-3-yl}acetic acid), see: Paneth (1995 ▶); McIntyre *et al.* (2001 ▶); Abou-Ghannam *et al.* (2012 ▶). For the synthesis and reactions of indomethacin with other non-steroidal anti-inflammatory mol­ecules, see: Mohamed *et al.* (2012 ▶).
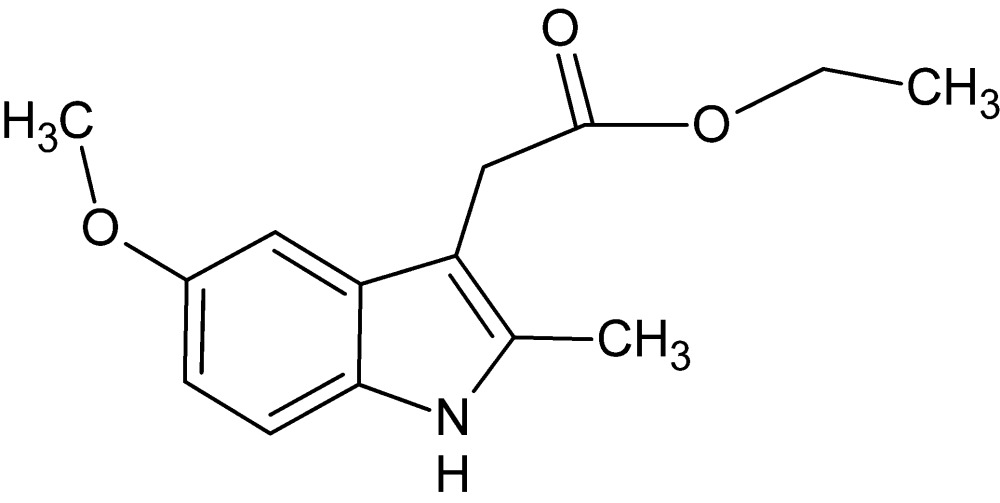


## Experimental

### 

#### Crystal data

C_14_H_17_NO_3_
*M* *_r_* = 247.29
Monoclinic, 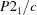

*a* = 7.8117 (5) Å
*b* = 17.1953 (12) Å
*c* = 9.9003 (7) Å
β = 106.756 (1)°
*V* = 1273.39 (15) Å^3^
*Z* = 4
Mo *K*α radiation
μ = 0.09 mm^−1^
*T* = 150 K
0.23 × 0.21 × 0.06 mm

#### Data collection

Bruker SMART APEX CCD diffractometer
Absorption correction: multi-scan (*SADABS*; Bruker, 2013 ▶) *T*_min_ = 0.84, *T*_max_ = 1.00
22947 measured reflections
3374 independent reflections
2889 reflections with *I* > 2σ(*I*)
*R*_int_ = 0.043

#### Refinement

*R*[*F* ^2^ > 2σ(*F* ^2^)] = 0.044
*wR*(*F* ^2^) = 0.117
*S* = 1.08
3374 reflections
170 parameters
H atoms treated by a mixture of independent and constrained refinement
Δρ_max_ = 0.31 e Å^−3^
Δρ_min_ = −0.24 e Å^−3^

### 

Data collection: *APEX2* (Bruker, 2013 ▶); cell refinement: *SAINT* (Bruker, 2013 ▶); data reduction: *SAINT*; program(s) used to solve structure: *SHELXS97* (Sheldrick, 2008 ▶); program(s) used to refine structure: *SHELXL97* (Sheldrick, 2008 ▶); molecular graphics: *ORTEP-3 for Windows* (Farrugia, 2012 ▶) and *DIAMOND* (Brandenburg & Putz, 2012 ▶); software used to prepare material for publication: *WinGX* (Farrugia, 2012 ▶) and *PLATON* (Spek, 2009 ▶).

## Supplementary Material

Crystal structure: contains datablock(s) global, I. DOI: 10.1107/S1600536813018618/su2618sup1.cif

Structure factors: contains datablock(s) I. DOI: 10.1107/S1600536813018618/su2618Isup2.hkl

Supplementary material file. DOI: 10.1107/S1600536813018618/su2618Isup3.cml

Additional supplementary materials:  crystallographic information; 3D view; checkCIF report

## Figures and Tables

**Table 1:** Hydrogen-bond geometry (Å, °) *Cg*1 is the centroid of the C1–C6 ring.

*D*—H⋯*A*	*D*—H	H⋯*A*	*D*⋯*A*	*D*—H⋯*A*
N1—H1⋯O2^i^	0.914 (18)	2.013 (18)	2.8987 (14)	162.7 (16)
C7—H7*B*⋯O2^ii^	0.98	2.57	3.4271 (18)	146
C13—H13*A*⋯O1^iii^	0.99	2.55	3.4236 (16)	148
C7—H7*A*⋯*Cg*1^iv^	0.98	2.99	3.9550 (16)	169

Symmetry codes: (i) 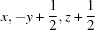; (ii) 
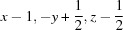
; (iii) 
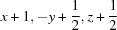
; (iv) 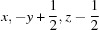.

**Fig. 1.**
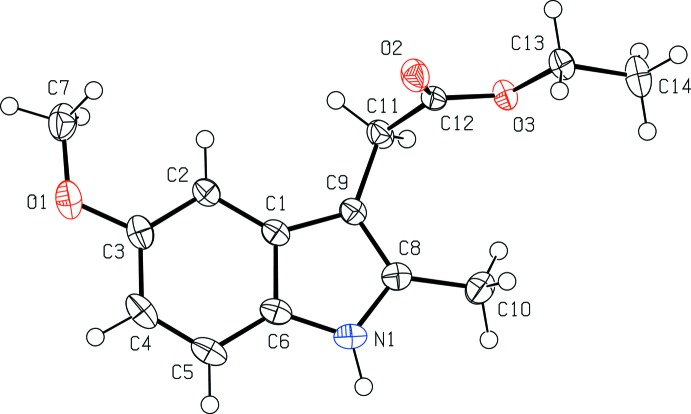
The molecular structure of the title molecule, with the atom numbering scheme. Displacement ellipsoids are drawn at the 50% probability level.

**Fig. 2.**
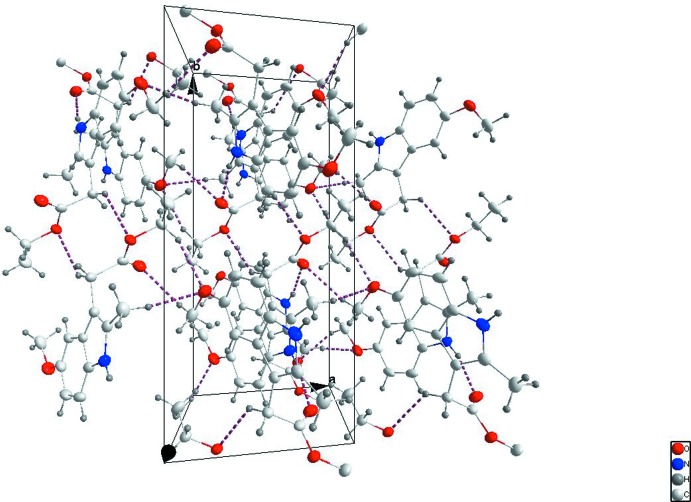
A perspective view along the c axis of the crystal packing of the title compound. The hydrogen bonds are shown as dashed lines - see Table 1 for details.

## supplementary crystallographic information

### Comment

Indomethacin, chemically named 2-{1-[(4-chlorophenyl)carbonyl]-5-methoxy-2-methyl-1*H*-indol-3-yl}acetic acid is a non-steroidal drug (NSAID) and is commonly used as an anti-inflammatory drug by inhibiting cyclooxygenase (COX) 1 and 2 enzymes. It also clinically used as a tocolytic agent to delay premature labor (preterm birth; PTB), reduce amniotic fluid in polyhydramnios, and to close patent ductus arteriosus (PDA). PTB is a major cause of neonatal morbidity and mortality worldwide (Paneth, 1995; McIntyre *et al.*, 2001; Abou-Ghannam *et al.*, 2012). In view of these facts and as part of our ongoing study incorporating NSAID's as a substructure in the synthesis of potential bio-active pharmacophors (Mohamed *et al.*, 2012), indomethacin has been hydrolysed during its esterification in acidic medium with ethanol to afforded the title corresponding ethyl ester.

In the title compound, Fig. 1, the nine-membered 1*H*-indole ring system (N1/C1–C6/C8/C9) is essentially planar with a maximum deviation of 0.019 (1) Å for N1. The C2–C3–O1–C7, C1–C9–C8–C10, C1–C9–C11–C12, C9–C11–C12–O2, C11–C12–O3–C13 and C12–O3–C13–C14 torsion angles are -6.53 (18), -177.77 (12), 117.42 (13), -69.51 (15), 177.50 (9) and 171.98 (10)°, respectively.

In the crystal, molecules are linked via N-H···O hydrogen bonds forming chains along [001]. These chains are linked via C-H···O hydrogen bonds and C-H···π interactions forming a two-dimensional network lying parallel to the ac plane (Fig. 2 and Table 1).

### Experimental

A mixture of 0.03 mol indomethacin (10.57 g m) in 150 ml of absolute ethanol and 6 ml of concentrated H_2_SO_4_ was refluxed for 6 h. The mixture was cooled to room temperature and neutralized with NaHCO_3_ solution. The ester was separated as an organic layer, washed with water and extracted with diethyl ether (3 × 50mL). The combined ether layers were dried over MgSO_4_, filtered and left for 3–4 days until brown crystals formed. The solid was collected and recrystallized from cyclohexane to give the pure ester as silver-coloured crystals (m.p. 347–350 K) suitable for X-ray diffraction. Spectroscopic data for the title compound are available in the archived CIF.

### Refinement

The C-bound H atoms were placed geometrically [C—H = 0.95 Å (aromatic H), C—H = 0.98 Å (methyl H) and 0.99 Å (methylene H)], and refined using a riding model with *U*_iso_(H) = 1.5*U*_eq_(C-methyl) and = 1.2*U*_eq_(C) for other H atoms. The N-bound H atom was located in a difference Fourier synthesis and freely refined [N1—H1 = 0.914 (18) Å].

### Crystal data

**Table:**

C_14_H_17_NO_3_	*F*(000) = 528
*M**_r_* = 247.29	*D*_x_ = 1.290 Mg m^−^^3^
Monoclinic, *P*2_1_/*c*	Mo *K*α radiation, λ = 0.71073 Å
Hall symbol: -P 2ybc	Cell parameters from 9989 reflections
*a* = 7.8117 (5) Å	θ = 2.4–29.1°
*b* = 17.1953 (12) Å	µ = 0.09 mm^−^^1^
*c* = 9.9003 (7) Å	*T* = 150 K
β = 106.756 (1)°	Plate, colourless
*V* = 1273.39 (15) Å^3^	0.23 × 0.21 × 0.06 mm
*Z* = 4	

### Data collection

**Table:**

Bruker SMART APEX CCD diffractometer	3374 independent reflections
Radiation source: fine-focus sealed tube	2889 reflections with *I* > 2σ(*I*)
Graphite monochromator	*R*_int_ = 0.043
Detector resolution: 8.3660 pixels mm^-1^	θ_max_ = 29.1°, θ_min_ = 2.4°
φ and ω scans	*h* = −10→10
Absorption correction: multi-scan (*SADABS*; Bruker, 2013)	*k* = −23→23
*T*_min_ = 0.84, *T*_max_ = 1.00	*l* = −13→13
22947 measured reflections	

### Refinement

**Table:**

Refinement on *F*^2^	Primary atom site location: difference Fourier map
Least-squares matrix: full	Secondary atom site location: inferred from neighbouring sites
*R*[*F*^2^ > 2σ(*F*^2^)] = 0.044	Hydrogen site location: difference Fourier map
*wR*(*F*^2^) = 0.117	H atoms treated by a mixture of independent and constrained refinement
*S* = 1.08	W = 1/[Σ^2^(*F*_o_^2^) + (0.0539*P*)^2^ + 0.3155*P*] where *P* = (*F*_o_^2^ + 2*F*_c_^2^)/3
3374 reflections	(Δ/σ)_max_ < 0.001
170 parameters	Δρ_max_ = 0.31 e Å^−^^3^
0 restraints	Δρ_min_ = −0.24 e Å^−^^3^

### Special details

**Table:**

Experimental. Spectroscopic data for the title compound: IR (KBr cm^-1^): (C=O ester 1728), (NH 3317), (C—H aliphatic, 2833–2924), (C—H, Ar, 2975–3002). ^1^*H*-NMR: (DMSO-D_6_) δ at 1.3(t, 3H, CH_3_ of ethyl group), 4.0(q, 2H, –CH_2_ aliphatic in ethyl group), 2.3(s, 3H, CH3), 3.4(s, –CH_2_), 3.7(s, 3H, –OCH_3_), 10.8(s, 1H, –NH), 6.8(s, 1H, Ar), 6.6(d, 1H, Ar), 7.2(d, 1H, Ar). ^13^C-NMR: 171 (C=O ester), 11(CH_3_ in indole), 14(CH_3_ of ethyl group), 29(–CH_2_), 55 (–OCH_3_), 59(–CH_2_ of ethyl group). 99, 103, 109, 110, 128, 129, 133,152 (8 C, aromatics). There are two signals at 29 and 59 p.p.m. oriented downward in the DEPT spectrum confirming the existence of two –CH_2_ groups.
Geometry. Bond distances, angles *etc*. have been calculated using the rounded fractional coordinates. All su's are estimated from the variances of the (full) variance-covariance matrix. The cell e.s.d.'s are taken into account in the estimation of distances, angles and torsion angles
Refinement. Refinement on *F*^2^ for ALL reflections except those flagged by the user for potential systematic errors. Weighted *R*-factors *wR* and all goodnesses of fit *S* are based on *F*^2^, conventional *R*-factors *R* are based on *F*, with *F* set to zero for negative *F*^2^. The observed criterion of *F*^2^ > σ(*F*^2^) is used only for calculating -*R*-factor-obs *etc*. and is not relevant to the choice of reflections for refinement. *R*-factors based on *F*^2^ are statistically about twice as large as those based on *F*, and *R*-factors based on ALL data will be even larger.

### Fractional atomic coordinates and isotropic or equivalent isotropic displacement parameters (Å^2^)

**Table:**

	*x*	*y*	*z*	*U*_iso_*/*U*_eq_	
O1	0.17116 (13)	0.36046 (6)	−0.24184 (10)	0.0376 (3)	
O2	0.75724 (12)	0.08270 (5)	−0.02263 (9)	0.0312 (3)	
O3	0.75501 (11)	0.01325 (4)	0.16773 (9)	0.0251 (2)	
N1	0.65214 (13)	0.29386 (6)	0.27056 (11)	0.0264 (3)	
C1	0.47230 (14)	0.25588 (6)	0.05968 (12)	0.0216 (3)	
C2	0.34868 (15)	0.26677 (6)	−0.07390 (13)	0.0240 (3)	
C3	0.29070 (16)	0.34165 (7)	−0.11378 (13)	0.0282 (3)	
C4	0.35262 (18)	0.40558 (7)	−0.02372 (15)	0.0336 (4)	
C5	0.47322 (17)	0.39589 (7)	0.10729 (15)	0.0309 (3)	
C6	0.53295 (15)	0.32062 (6)	0.14800 (13)	0.0245 (3)	
C7	0.09045 (18)	0.29785 (9)	−0.33106 (15)	0.0367 (4)	
C8	0.66375 (15)	0.21415 (7)	0.26458 (12)	0.0243 (3)	
C9	0.55670 (14)	0.18869 (6)	0.13595 (12)	0.0220 (3)	
C10	0.77677 (16)	0.16917 (8)	0.38672 (13)	0.0312 (4)	
C11	0.52395 (15)	0.10556 (6)	0.08854 (13)	0.0258 (3)	
C12	0.68958 (14)	0.06698 (6)	0.06967 (12)	0.0224 (3)	
C13	0.91945 (15)	−0.02529 (7)	0.16271 (13)	0.0272 (3)	
C14	0.98064 (19)	−0.07392 (8)	0.29348 (16)	0.0395 (4)	
H1	0.700 (2)	0.3250 (10)	0.347 (2)	0.052 (5)*	
H2	0.30620	0.22390	−0.13500	0.0290*	
H4	0.31030	0.45630	−0.05410	0.0400*	
H5	0.51440	0.43900	0.16800	0.0370*	
H7A	0.18300	0.26750	−0.35610	0.0550*	
H7B	0.00600	0.31840	−0.41700	0.0550*	
H7C	0.02660	0.26440	−0.28160	0.0550*	
H10A	0.72640	0.11700	0.38710	0.0470*	
H10B	0.77960	0.19600	0.47470	0.0470*	
H10C	0.89850	0.16500	0.37860	0.0470*	
H11A	0.42780	0.10380	−0.00200	0.0310*	
H11B	0.48250	0.07610	0.15910	0.0310*	
H13A	1.01170	0.01370	0.16000	0.0330*	
H13B	0.89690	−0.05840	0.07760	0.0330*	
H14A	1.00740	−0.04020	0.37670	0.0590*	
H14B	1.08850	−0.10270	0.29240	0.0590*	
H14C	0.88610	−0.11070	0.29690	0.0590*	

### Atomic displacement parameters (Å^2^)

**Table:**

	*U*^11^	*U*^22^	*U*^33^	*U*^12^	*U*^13^	*U*^23^
O1	0.0347 (5)	0.0333 (5)	0.0417 (5)	0.0089 (4)	0.0059 (4)	0.0110 (4)
O2	0.0383 (5)	0.0288 (4)	0.0283 (4)	0.0066 (4)	0.0124 (4)	0.0065 (3)
O3	0.0260 (4)	0.0219 (4)	0.0277 (4)	0.0049 (3)	0.0084 (3)	0.0047 (3)
N1	0.0275 (5)	0.0268 (5)	0.0265 (5)	−0.0049 (4)	0.0104 (4)	−0.0073 (4)
C1	0.0206 (5)	0.0194 (5)	0.0273 (5)	−0.0005 (4)	0.0111 (4)	−0.0010 (4)
C2	0.0233 (5)	0.0217 (5)	0.0283 (6)	0.0017 (4)	0.0093 (4)	−0.0001 (4)
C3	0.0258 (5)	0.0269 (6)	0.0340 (6)	0.0052 (4)	0.0122 (5)	0.0067 (5)
C4	0.0348 (6)	0.0200 (5)	0.0503 (8)	0.0051 (5)	0.0191 (6)	0.0048 (5)
C5	0.0352 (6)	0.0196 (5)	0.0432 (7)	−0.0024 (5)	0.0198 (6)	−0.0048 (5)
C6	0.0251 (5)	0.0222 (5)	0.0299 (6)	−0.0027 (4)	0.0140 (4)	−0.0039 (4)
C7	0.0297 (6)	0.0455 (8)	0.0335 (7)	0.0111 (5)	0.0071 (5)	0.0055 (6)
C8	0.0227 (5)	0.0268 (5)	0.0255 (5)	−0.0017 (4)	0.0104 (4)	−0.0018 (4)
C9	0.0199 (5)	0.0205 (5)	0.0265 (5)	−0.0002 (4)	0.0080 (4)	−0.0006 (4)
C10	0.0282 (6)	0.0394 (7)	0.0249 (6)	0.0023 (5)	0.0058 (5)	0.0005 (5)
C11	0.0216 (5)	0.0191 (5)	0.0345 (6)	−0.0002 (4)	0.0047 (4)	−0.0003 (4)
C12	0.0239 (5)	0.0164 (5)	0.0243 (5)	−0.0006 (4)	0.0027 (4)	−0.0016 (4)
C13	0.0244 (5)	0.0247 (5)	0.0323 (6)	0.0060 (4)	0.0080 (5)	0.0028 (5)
C14	0.0364 (7)	0.0369 (7)	0.0444 (8)	0.0134 (6)	0.0106 (6)	0.0145 (6)

### Geometric parameters (Å, º)

**Table:**

O1—C3	1.3789 (16)	C11—C12	1.5128 (16)
O1—C7	1.4198 (18)	C13—C14	1.4990 (19)
O2—C12	1.2108 (15)	C2—H2	0.9500
O3—C12	1.3309 (13)	C4—H4	0.9500
O3—C13	1.4590 (15)	C5—H5	0.9500
N1—C6	1.3782 (16)	C7—H7A	0.9800
N1—C8	1.3760 (16)	C7—H7B	0.9800
N1—H1	0.914 (18)	C7—H7C	0.9800
C1—C2	1.4072 (17)	C10—H10A	0.9800
C1—C6	1.4105 (15)	C10—H10B	0.9800
C1—C9	1.4327 (15)	C10—H10C	0.9800
C2—C3	1.3841 (16)	C11—H11A	0.9900
C3—C4	1.4102 (18)	C11—H11B	0.9900
C4—C5	1.376 (2)	C13—H13A	0.9900
C5—C6	1.3953 (16)	C13—H13B	0.9900
C8—C9	1.3779 (16)	C14—H14A	0.9800
C8—C10	1.4914 (17)	C14—H14B	0.9800
C9—C11	1.5033 (15)	C14—H14C	0.9800
			
C3—O1—C7	117.11 (11)	C5—C4—H4	119.00
C12—O3—C13	116.57 (9)	C4—C5—H5	121.00
C6—N1—C8	109.32 (10)	C6—C5—H5	121.00
C6—N1—H1	123.0 (11)	O1—C7—H7A	109.00
C8—N1—H1	127.1 (11)	O1—C7—H7B	109.00
C2—C1—C6	119.63 (10)	O1—C7—H7C	109.00
C6—C1—C9	106.77 (10)	H7A—C7—H7B	109.00
C2—C1—C9	133.59 (10)	H7A—C7—H7C	109.00
C1—C2—C3	118.12 (10)	H7B—C7—H7C	109.00
O1—C3—C2	124.03 (11)	C8—C10—H10A	109.00
O1—C3—C4	114.61 (11)	C8—C10—H10B	109.00
C2—C3—C4	121.36 (12)	C8—C10—H10C	109.00
C3—C4—C5	121.23 (11)	H10A—C10—H10B	109.00
C4—C5—C6	117.76 (12)	H10A—C10—H10C	109.00
C1—C6—C5	121.90 (11)	H10B—C10—H10C	109.00
N1—C6—C5	130.45 (11)	C9—C11—H11A	109.00
N1—C6—C1	107.65 (9)	C9—C11—H11B	109.00
N1—C8—C9	109.04 (10)	C12—C11—H11A	109.00
N1—C8—C10	120.84 (11)	C12—C11—H11B	109.00
C9—C8—C10	130.11 (11)	H11A—C11—H11B	108.00
C1—C9—C8	107.18 (10)	O3—C13—H13A	110.00
C8—C9—C11	126.44 (10)	O3—C13—H13B	110.00
C1—C9—C11	126.24 (10)	C14—C13—H13A	110.00
C9—C11—C12	112.39 (10)	C14—C13—H13B	110.00
O2—C12—O3	123.10 (11)	H13A—C13—H13B	109.00
O2—C12—C11	124.77 (10)	C13—C14—H14A	110.00
O3—C12—C11	112.13 (10)	C13—C14—H14B	109.00
O3—C13—C14	106.78 (10)	C13—C14—H14C	110.00
C1—C2—H2	121.00	H14A—C14—H14B	109.00
C3—C2—H2	121.00	H14A—C14—H14C	109.00
C3—C4—H4	119.00	H14B—C14—H14C	109.00
			
C7—O1—C3—C2	6.53 (18)	C6—C1—C9—C8	−0.25 (13)
C7—O1—C3—C4	−173.81 (12)	C6—C1—C9—C11	175.68 (11)
C13—O3—C12—O2	2.08 (16)	C1—C2—C3—O1	179.49 (11)
C13—O3—C12—C11	−177.50 (9)	C1—C2—C3—C4	−0.15 (19)
C12—O3—C13—C14	171.98 (10)	O1—C3—C4—C5	−179.64 (13)
C8—N1—C6—C1	−2.16 (14)	C2—C3—C4—C5	0.0 (2)
C8—N1—C6—C5	177.77 (13)	C3—C4—C5—C6	0.3 (2)
C6—N1—C8—C9	2.03 (14)	C4—C5—C6—N1	179.64 (13)
C6—N1—C8—C10	−176.93 (11)	C4—C5—C6—C1	−0.4 (2)
C6—C1—C2—C3	−0.02 (17)	N1—C8—C9—C1	−1.07 (13)
C9—C1—C2—C3	178.39 (13)	N1—C8—C9—C11	−176.98 (11)
C2—C1—C6—N1	−179.74 (11)	C10—C8—C9—C1	177.77 (12)
C2—C1—C6—C5	0.33 (18)	C10—C8—C9—C11	1.9 (2)
C9—C1—C6—N1	1.47 (13)	C1—C9—C11—C12	117.42 (13)
C9—C1—C6—C5	−178.47 (12)	C8—C9—C11—C12	−67.42 (15)
C2—C1—C9—C8	−178.81 (13)	C9—C11—C12—O2	−69.51 (15)
C2—C1—C9—C11	−2.9 (2)	C9—C11—C12—O3	110.07 (11)

### Hydrogen-bond geometry (Å, º)

Cg1 is the centroid of the C1–C6 ring.

**Table:**

*D*—H···*A*	*D*—H	H···*A*	*D*···*A*	*D*—H···*A*
N1—H1···O2^i^	0.914 (18)	2.013 (18)	2.8987 (14)	162.7 (16)
C7—H7*B*···O2^ii^	0.98	2.57	3.4271 (18)	146
C13—H13*A*···O1^iii^	0.99	2.55	3.4236 (16)	148
C7—H7*A*···*Cg*1^iv^	0.98	2.99	3.9550 (16)	169

Symmetry codes: (i) *x*, −*y*+1/2, *z*+1/2; (ii) *x*−1, −*y*+1/2, *z*−1/2; (iii) *x*+1, −*y*+1/2, *z*+1/2; (iv) *x*, −*y*+1/2, *z*−1/2.
